# Exploring the emotional impact of axial Spondyloarthritis: a systematic review and thematic synthesis of qualitative studies and a review of social media

**DOI:** 10.1186/s41927-023-00351-w

**Published:** 2023-08-23

**Authors:** Nicky Wilson, Jia Liu, Qainat Adamjee, Sonya Di Giorgio, Sophia Steer, Jane Hutton, Heidi Lempp

**Affiliations:** 1 Department of Rheumatology, King’s College Hospital NHS Foundation Trust, London, UK; 2 Centre for Education, Faculty of Life Sciences & Medicine, King’s College London, London, UK; 3 GKT School of Medical Education, King’s College London, London, UK; 4 King’s College London Libraries & Collections, King’s College London, London, UK; 5 Department of Clinical Health Psychology, Liverpool University Hospitals NHS Foundation Trust, Liverpool, UK; 6 Department of Inflammation Biology, Centre for Rheumatic Diseases, School of Immunology and Microbial Sciences, Faculty of Life Sciences & Medicine, King’s College London, London, UK

**Keywords:** Axial Spondyloarthritis, Affect, Qualitative, Thematic synthesis

## Abstract

**Background:**

The psychological burden in people with inflammatory arthritis is substantial, yet little is known about the disease-related affect experienced by individuals with axial Spondyloarthritis (axial SpA). The aim of this study was to conduct a qualitative evidence synthesis and a review of social media to explore the emotional impact of living with axial SpA.

**Methods:**

We searched nine databases for studies reporting qualitative data about participants’ emotional experience of living with axial SpA. In addition, we searched social media platforms for posts from people with axial SpA based in the UK that offered insights into emotional responses to living with the condition. We employed a thematic approach to synthesise the data.

**Results:**

We included 27 studies (1314 participants; 72% men) in our qualitative evidence synthesis and developed seven descriptive themes from the data: 1) delayed diagnosis: a barrier to emotional wellbeing; 2) disruptive symptoms: a source of mood swings; 3) work disability: a loss of self-esteem; 4) obstacles in interpersonal relationships: a trigger of distress; 5) taking up exercise: personal pride or unwelcomed reminders; 6) anti-TNF therapy: hope reignited despite concerns and 7) a journey of acceptance: worry mixed with hope. Posts extracted from social media fora (537; 48% from women) for the most part supported the seven themes. One additional theme—COVID-19, uncertainty and anxiety during the pandemic, was developed, reflecting common emotions expressed during the UK’s first wave of the coronavirus pandemic.

**Conclusion:**

This study highlights a preponderance of negative affect experienced by people living with axial SpA, conditioned through existing and anticipated symptoms, failed expectations, and lost sense of self. Given the bidirectional relationships between negative emotions and inflammation, negative emotions and perceptions of pain, and the influence of affect in self-care behaviours, this finding has important implications for treatment and management of people with axial SpA.

**Supplementary Information:**

The online version contains supplementary material available at 10.1186/s41927-023-00351-w.

## Background

Axial Spondyloarthritis, incorporating Ankylosing Spondylitis (AS) and non-radiographic axial Spondyloarthritis (n-r axial SpA), is a type of inflammatory arthritis that starts commonly in early adult life [1]. It carries a considerable mental health burden; incidence estimates suggest risk of depression is 51% higher in people with AS compared to individuals without AS [2], while in those newly diagnosed, the risk is higher still – twofold that of the general population [3]. Furthermore, being diagnosed with axial Spondyloarthritis (axial SpA) increases risk of deliberate self-harm by 59% compared to non-diagnosed individuals [4].

In addition to those diagnosed with mental ill health, such as depression, many more people with axial SpA experience symptoms of anxiety and depression that fall short of diagnostic cut points yet exceed levels experienced by the general population [5, 6]. A survey of over 2000 people from 13 countries in Europe highlighted nearly two thirds (60.7%) were at risk of mental illness when assessed for psychological distress using the 12-item General Health Questionnaire [5].

Further insights into the mental health burden in people with axial SpA can be gleaned from qualitative research that captures affective experience [7, 8]. Affective experience includes emotions and moods, which differ in duration and specificity but share dimensions of valence, motivation and arousal [8]. While the linguistic expression of emotions and moods are similar, emotions are short term felt episodes that occur in response to objects or events of significance to one’s allostatic state. In contrast, moods are more prolonged and diffuse and lack a specific focus [9]. Both however are highly influential in mental and physical health [10, 11].

Several qualitative evidence syntheses exploring the lived experiences of people with inflammatory arthritis highlight the toll these conditions take on mental wellbeing [12–14]. For example, Toye and colleagues [12] bring to light the distress felt by people with rheumatoid arthritis (RA) from loss of sense of self because of role changes associated with their inflammatory arthritis and delayed diagnosis. Stewart et al. [13] captures experiences of shame and embarrassment borne by people experiencing a gout flare, while Sumpton et al. [14] illuminates the anger felt among people with psoriatic arthritis (PsA) about the perceived lack of attention from others to their emotional wellbeing. To our knowledge, there is no published qualitative evidence synthesis capturing the affective experiences of people with axial SpA. The aim of this study was to undertake a qualitative evidence synthesis and a review of social media to explore the emotional impact of living with axial SpA. Our decision to undertake a review of social media alongside a synthesis of qualitative research was influenced by knowledge of a likely gender bias in published studies, as historically AS has been considered predominantly a male disease [15].

## Methods

### Qualitative evidence synthesis

A priori study protocol was registered in the international prospective register of systematic reviews (CRD 42020162478) and search strategies (see Additional file 1) developed in collaboration with a health librarian. Nine electronic databases (MEDLINE, EMBASE, CINAHL, PsycINFO, Web of Science, Scopus, Cochrane systematic reviews, EThOS and OpenGrey) were searched from inception to the 3^rd^ week in December 2019 for relevant studies. Two reviewers (NW and HL) independently screened record titles and abstracts for studies meeting the pre-determined inclusion criteria: (i) Adults ≥ 16 years with axial SpA (AS or n-r axial SpA), (ii) reporting qualitative data about participants emotional experience of axial SpA and iii) published in English or German. Mixed methods studies were included if qualitative data could be analysed separately. The first 10% of records were screened in duplicate and inter-assessor reliability was calculated using Cohen’s Kappa (*K* = 0.44). Following this, both reviewers discussed and clarified the selection criteria before continuing screening independently. Due to the impact of the COVID-19 pandemic, the review was temporarily suspended, and an up-to-date search was conducted the week commencing 15th March 2021. Titles and abstracts identified in this search were screened by one reviewer (NW).

Full text review of included studies and generation of a final list was undertaken independently by two reviewers (NW and JL). Disagreements were resolved by discussion and involvement of a third reviewer (HL) as necessary. Data from included studies (year of publication, country, study source, study design, participant characteristics including disease or symptom duration, and method of qualitative data analysis) were extracted using a pre-piloted data extraction template. Reference lists of selected studies were screened for additional studies.

Quality appraisal was undertaken independently by two authors (NW and JL) using the Critical Appraisal Skills Programme (CASP) tool for qualitative peer reviewed evidence [16] and the Authority, Accuracy, Coverage, Objectivity, Date, Significance (AACODS) checklist for grey literature [17]. We supplemented the CASP tool with two additional questions as suggested by Long et al. [18]. First, a question to clarify reporting of the theoretical underpinnings of a study and second, a ‘somewhat or partly’ category to the yes, can’t tell or no tool response options. For mixed methods studies, appraisal focussed on the qualitative arm. Judgement about the trustworthiness of a study (strong, moderate, weak) was based on two questions: i) was the recruitment strategy appropriate to the research aims and ii) was data analysis sufficiently rigorous, or for grey literature publications, was the study significant in the context of relevant research. A third reviewer (HL) moderated when disagreements arose. No studies were excluded based on the quality appraisal.

Manuscripts of included studies were uploaded to NVivo 12 (QSR International), a qualitative computer software programme. A thematic synthesis [19, 20] was employed. Text in the abstract, results, discussion, and supplementary files that captured (i) participant emotional experiences related to living with axial SpA, (ii) the source and (iii) the context of those emotions, were coded. First, we coded high quality studies providing thick (ample, rich) or moderately thick descriptive data about emotional experiences. Data relating to emotional experiences or affect were coded line by line. Using the method of constant comparison [21], similar codes were organised into categories. Subsequently, we translated codes from medium quality studies with thick or moderately thick descriptive data into the extrapolated categories and created new constructs as necessary. Finally, weak quality studies and studies with thin data (i.e., limited qualitative evidence) were coded and any new codes assimilated into the developing framework. From these categories we developed descriptive themes relating to the emotional impact of axial SpA. These themes, along with other literature about experiences of living with chronic illness informed the development of analytical themes. Reporting of our review and synthesis is in accordance with the ENhancing Transparency in REporting the synthesis of qualitative research (ENTREQ) statement [22] (Additional file 3).

### Social media review

A search of social media (including forums of the National Axial Spondyloarthritis Society (NASS), Twitter, Facebook, Instagram and public blogs) for posts containing data about the emotional experiences of people living with axial SpA based in the UK, was undertaken by QA. Written permission to access forums was obtained and fora were searched from inception to 28^th^ June 2020. Posts were selected if they reflected concepts relating to the emotional impact of living with axial SpA or described barriers or facilitators to emotional well-being. Plutchik’s emotion wheel [23] guided identification of relevant posts, while posts from individuals based outside of the UK were excluded. Selected posts were extracted, de-identified and uploaded to NVivo 12 for organisation and coding. Coding and categorising were undertaken by QA and NW independently. Themes were developed by NW and the data were applied to challenge, support and extend the thematic synthesis. Posts analysed are not quoted verbatim in this study, as permissions were not sought from authors to use their data.

## Results

### Qualitative evidence synthesis

**Fig. 1:**
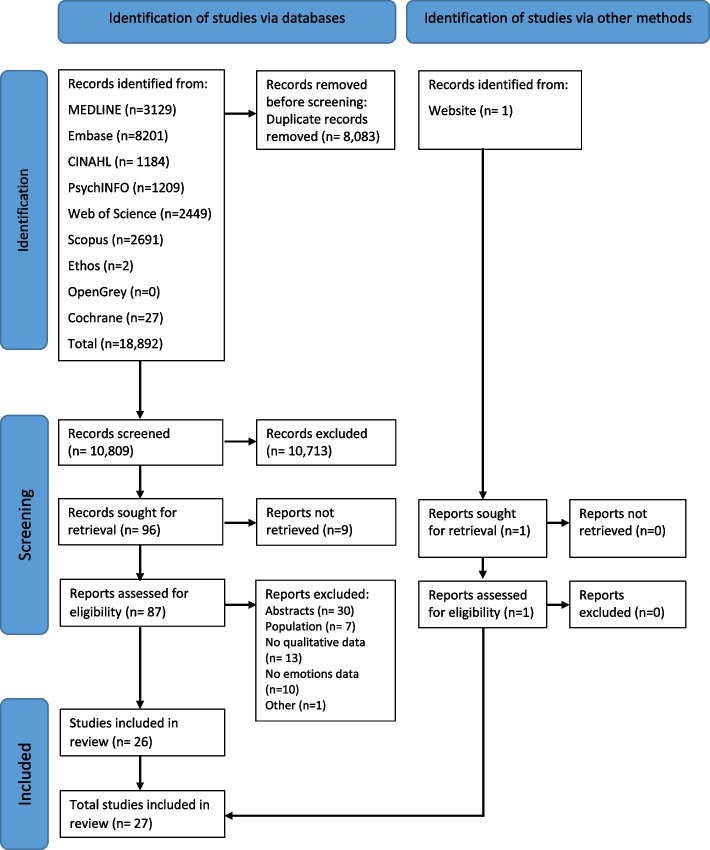
Flow diagram of studies included in the systematic review

The database searches yielded 18,892 records. One additional record was identified through other sources. Eighty-eight reports were assessed for eligibility, from which 27 studies were included in the review (Fig. 1).

Studies were published between 1995 and 2020 and were conducted in the UK (14), Europe (9), the Americas (3) and Asia (1). Study designs included 21 qualitative (one was an autobiographical account of living with AS [24]) and six mixed methods studies, including one doctoral thesis [25]. Most studies were cross-sectional although Thompson [25] conducted serial interviews with newly diagnosed patients with AS.

Study populations included participants with AS (*n* = 1220) [24–45], axial SpA (*n* = 76) [46–49] and n-r axial SpA (*n* = 18) [50]. Nearly three quarters of participants (*n* = 948, 72%) were men and most had established disease. When stated, 189 participants were on treatment with biologic therapy. Two studies [26, 27] included focus groups with mixed populations with inflammatory arthritis but did not specify the number of participants with AS. Study foci included experiences of symptoms, delayed diagnosis, work, physical activity, relationships, treatment with anti-TNFα therapy and educational needs (see Table 1.). We did not identify any studies focussed specifically on the emotional or psychological experiences of people with axial SpA.

**Table 1:** Overview of included studies

**First author (year), Country, ** **Study source**	**Study design**	**Study population**	**Method of analysis**	**Study summary**
James (2009) [24], UK	Autobiographical narrative	N=1 with AS (Men 100%) Man in his 50s Symptom duration: since 11 years	N/A	A personal account of living life with AS
Thompson (2011) [25], UK, NASS and three rheumatology departments.	Mixed methods study including qualitative focus group and interviews	N=8 with AS^a^ (Men 50%) Age: range 28-71 years Disease duration: NS	Content analysis	To understand the educational needs of people with AS
		N=22 with AS^b^ (Men 77%) Age: 38.7 years (mean) Disease duration: NS		
Barlow (1999) [26], UK, NASS and volunteers from research centre or Arthritis Care	Mixed method study including questionnaire with open-ended questions and qualitative focus groups	N=145 with AS (Men 68%)^c^ Age: 51.4 years (mean) Disease duration: 25.05 years (mean) N=NS with arthritis, mostly RA and AS^a^ Age: NS Disease duration: NS	Content analysis	To examine perceptions of parenting in mothers, fathers and grandparents with arthritis
Haugli (2004) [27], Norway, In-patients in a rehabilitation centre	Qualitative focus groups	N= 12 with RA or AS^c^ (Male 33%) Age: range 20-80 years Disease duration: range 1-30 years	Thematic analysis	To explore the doctor-patient relationship in patients with rheumatic disease
Madsen (2015) [28], Denmark, Outpatient rheumatology clinic	Qualitative interviews	N=13 with AS (Men 100%) Age: 44 years (median) Disease duration: 12 years (median)	Content analysis	To understand men’s experiences of living with AS
Primholdt (2017) [29], Denmark, Rheumatology hospital department	Qualitative interviews	N=5 with AS (Men 100%) Age: range 21-37 years Disease duration: range 1-5 years	Meaning condensation	To explore younger men’s experiences of living with AS
Stockdale (2008) [30], UK Rheumatology department	Qualitative interviews	N=8 with AS (Men 100%) Age: 46.1 years (mean) Disease duration: 18.8 years	Thematic analysis	To explore the impact of taking anti TNFα therapy in patients with AS
Bagcivan (2015) [31], Turkey, Hospital rheumatology outpatient clinic	Qualitative interviews	N=23 with AS (Men 70%) Age: 29.65 years (mean) Disease duration: 5.39 years (mean)	Thematic analysis	To explore the experience of pain in patients with AS
Davies (2013) [32], UK, Patient population in Wales	Qualitative focus groups	N=14 with AS (Men 50%) Age: 53 years (mean) Disease duration: 29 years (mean)	Thematic analysis	To explore the effect of fatigue in patients with AS
Mengshoel (2010) [33], Norway, Rehabilitation center or self-referral	Qualitative interviews	N=12 with AS (Men 33%) Age: range 30-59 years Disease duration: range 6 months -36 years	Thematic analysis	To examine the nature of fatigue and how it is managed in patients with AS
Brophy (2002) [34], UK, Residential management programme	Qualitative group discussion	N=214 with AS (Men 79%) Age: 47 years (mean) Disease duration: 25 years (mean)	Content analysis	To examine patient perspectives of disease flare
Cury (1995) [35], Brazil, Outpatient rheumatology clinic	Qualitative focus groups	N=15 with AS (Men 100%) Age 32.6 years (mean) Disease duration: 13.4 years (mean)	Content analysis	Understanding of the origin and management of AS
Lacaille (2007) [36], Canada, Arthritis treatment programme and rheumatology private practices	Qualitative focus groups	N=5/36 with AS (NS) Age: NS Disease duration: NS	Descriptive analysis	To understand patients experiences at work in relation to their inflammatory arthritis
Barlow (2001) [37], UK, Rheumatology outpatient clinics and members of NASS	Mixed methods study including questionnaire with open-ended questions and qualitative interviews	N=133 with AS (Men 73%) Age: 49 years (mean) Disease duration: 28 years (mean)	Thematic analysis	To examine the meaning and perceived impact of work disability in patients with AS
Farren (2013) [38], UK, Physiotherapy or MDT AS clinics	Qualitative diary-interviews	N=10 with AS (Men 60%) Age: range 28-66 years Disease duration: range 10-35 years	Framework analysis	To understand patient experiences of fatigue
Hamilton-West (2009) [39], UK, NASS	Open-ended questoinnaire	N=68 with AS (Men 66%) Age: 52 years (mean) Disease duration: 15 years (mean)	Content analysis	To understand patient perspectives of the implications of AS.
O’Dwyer (2016) [40], UK, Rheumatology outpatient clinic and national patient support groups	Qualitative interviews	N=17 with AS (Men 53%) Mean age 39.3 (SD 9.5) Symptom duration: 12 years	Thematic analysis	To understand patients with AS attitudes towards physical activity and exercise
Stockdale (2014) [41], UK, Hospital rheumatology department	Qualitative interviews	N=20 with AS (Men 90%) Age: range 25-75 years Disease duration: range 3-36 years	Thematic network analysis	To explore the effects of anti-TNFα medication on exercise behaviour in patients with AS
Cinar (2014) [42], Turkey, Hospital rheumatology outpatient clinic	Mixed methods study using a descriptive questionnaire	N=101 with AS (Men 94%) Age: 36.55 years (mean) Disease duration: 12.36 years (mean)	Thematic analysis	To explore views about anti TNFα therapy in patients with AS
Connolly (2019) [43], Ireland, Hospital AS clinic	Mixed methods study including qualitative interviews	N=19 with AS (Men 68%) Age: 46.7 years (mean) Disease duration: NS	Content analysis	To explore experiences of fatigue and management strategies in patients with AS.
Brophy (2013) [44], UK, Residents in one Health Board in Wales	Mixed methods study using a questionnaire with open-ended questions	N=348 with AS^e^ (Men 71%) Age: NS Disease duration: NS	Thematic analysis	To explore patient experience of fatigue and personal management strategies.
Boonen (2009) [45], The Netherlands, Outpatient rheumatology clinic	Qualitative focus groups	N=19 with AS (Men 74%) Age: 54 years (mean) Disease duration: 18.7 years (mean)	Meaning condensation	To explore concepts important for functioning in patients with AS
Martindale (2014) [46], UK, Two rheumatology departments.	Qualitative interviews	N= 10 with axSpA (Men 70%) Age: 40.2 years (mean) Symptom duration: 10.1 years (mean)	Interpretive phenomenological analysis	To explore the journey to diagnosis of people with axSpA
Berenbaum (2014) [47], France, Hospital and community-based rheumatology services.	Qualitative interviews	N=23/25 with axSpA (Men 48%)^d^ Age: ≥35 years (28%); 36 to 45 years (28%); 46 to 55 years (24%); >55 years (20%) Disease duration: <2 years (24%), 2-5 years (24%), 6-10 years (24%), > 10 years (28%)	Thematic analysis	To explore beliefs and fears about RA and SpA and about treatment of these diseases
Raybone (2019) [48], UK, axSpA-specific charity	Qualitative interviews	N=9 with axSpA (Male 33%) Age: 42.4 years (mean) Disease duration: NS	Thematic analysis	To explore the impact of AS on couple relationships
Kwan (2019) [49], Singapore, Hospital registry	Qualitative focus groups	N=34 with axSpA (Men 59%) Age: 41 years (median) Disease duration: 11 years (median)	Thematic analysis	To explore Quality-of-Life domains
Hwang (2020) [50], United States, Two clinic sites and a prior qualitative study	Qualitative interviews	N=18 with nr-axSpA (Men 33%) Age: 46 years (Median) Disease duration: range 3 months -15 years	Content analysis	To explore the relevance of concepts measured by the Ankylosing Spondylitis Quality of Life (ASQoL) instrument

*Abbreviations*: AS, Ankylosing spondylitis; NASS, National Axial Spondyloarthritis Society; SpA, Spondyloarthritis; RA, Rheumatoid Arthritis; NS, not specified; axSpA, axial Spondyloarthritis; nr-axSpA, non-radiographic axial Spondyloarthritis

^a^ Participated in focus group

^b^ Participated in qualitative interviews

^c^ Participated in questionnaire with open-ended questions

^d^ The number of participants with axial SpA was not specified

^e^ The total number of participants with AS that provided qualitative data was not specified

Following appraisal, 70% of the included studies were categorised as moderate to strong quality (see Additional file 2). Only 13 studies contained thick or moderately thick descriptions about participants affective experiences. Seven descriptive themes were generated through the synthesis: 1) delayed diagnosis: a barrier to emotional wellbeing; 2) disruptive symptoms: a source of mood swings; 3) work disability: a loss of self-esteem; 4) obstacles in interpersonal relationships: a trigger of distress; 5) taking up exercise: personal pride or unwelcomed reminders; 6) anti-TNF therapy: hope reignited despite concerns and 7) a journey of acceptance: worry mixed with hope. Themes are written in the first person, following the example of Toye et al. [12] and supported by example participant and author accounts (Table 2).

**Table 2:** Descriptive themes generated through synthesis of qualitative studies with example accounts

**Descriptive themes**	**Participants’ accounts**	**Authors’ accounts**
Delayed diagnosis: a barrier to emotional wellbeing	“But when it’s inside the body and it’s not presenting itself in any see-able way, you just can’t explain to people. To be honest, you get fed up with it, of trying to explain.” [46] “…but coming away with this diagnosis of just being out of shape disheartened me and I actually came out of the hospital upset with my free gym membership, which I was devastated about.” [46] “My GP was unsympathetic. He gave me the distinct impression that he thought I was some sort of malingerer, even though I resisted the idea of taking time off work.” [24]	They [participants] experienced limitations in their [physical abilities and had trouble accomplishing daily life tasks […]. This period [prior to diagnosis] was described as glum, long, uncomfortable and frustrating [28]. The delay in diagnosis was described as upsetting, distressing and disheartening. Feelings of anger, frustration about the wasted time, and disappointment were expressed. The lack of knowledge and control over what they [the participants] had suffered was evident, as were feelings of depression [46]. The time until diagnosis was filled with pain and worries about the future [29].
Disruptive symptoms: a source of mood swings	“Suddenly, without warning pain would strike and I felt immobile, helpless and frustrated.” [24] “I was great for 4 years and then I flared so badly I could barely walk to work or write. Lifting up a pencil was killing me. So you could be going along and everything is on track and then everything just falls apart.” [36] “I had so much pain in my buttocks that I could not get on my feet from where I was sitting … […]. I could not reach my pain medicine. I could not get to the telephone to call a friend. The windows were open, the room was getting cold, and I could not get up …I had never felt so helpless before. I cried.” [31] “AS often gives the sufferer a general feeling of being poorly or unwell. […]. Nerves are on edge and a more grumpy or aggressive mood is common. Tiredness and lethargy accompany this phase which tends to last a few days at a time.” [24]	During periods of remission …the fear of future disability would temporarily recede. However, when an exacerbation occurred, or if the treatment stopped working, initial fears were often reactivated. The variability in the course and presentation of the disease was …a major source of uncertainty and worry about the future [47]. Some [participants] described feeling guilty about how pain and fatigue made them more irritable and impatient in their relationships at work and at home [36]. The respondents said that times like these [a flare up] reminded them that they had an unpredictable disease. They had no power and felt helpless because they could do nothing to get relief or to predict what was likely to come in the future [33].
Work disability: a loss of self-esteem	“it’s frustrating but when you get frustrated you get annoyed because you have to go to work, you want to go to work. I enjoy work and you just can’t do the job and you end up getting, probably depressed.” [46] ‘It [fatigue] is sort of a quiet, silent thing that happens to you and you sit back at the end of the day and you think—I could have done that better, why didn’t I do that?” [36] “I am frustrated that I have no stamina, that I’m such a weakling. That I can’t just suck it up and then stay at work, and that I have to go home early” [29]. “I missed calls at night. I didn’t wake up …they had gone without me and then you feel as if you are letting people down.” [38]	There was a divergence of views regarding reactions to changed working lives. The majority believed that changes were negative and engendered depressed mood; frustration; bitterness; anger; mood swings; feelings of inadequacy; and loss of choice, independence, self-esteem, self-confidence, and job satisfaction [37]. The men experienced a sense of unworthiness if they could not manage a job, feeling that they did not live up to society’s expectations [28].
Obstacles in interpersonal relationships: a trigger of distress	“I was exhausted I was in a lot of pain, I was tetchy and we did separate for 12 months. […]. I was pushing and pushing away I think because I could not understand why he wanted to be with me …” [48] “I was a nightmare to live with …I couldn’t express myself, I couldn’t do anything because when you are not sleeping, because you are in such pain, you become a different man, you just do, you are not nice to be around and I wasn’t …I was a pig to live with […]” [30]. “In some social situations …I’m there, but I do not participate if I’m …not in form. …then I’m only sitting there and …seeming rather indifferent … That is really frustrating.” [33] “One or two [friends] have dropped out [no longer in contact] because they couldn’t really accept that you just couldn’t go out with them every weekend.” [28]	As children grew older the difficulties centered on the negative feelings of both parents and children when pre-arranged commitments had to be cancelled due to parents having a ‘bad day’ …described by one father as the ‘I let you down again syndrome’ [26]. …the men experienced that it was often difficult for their friends to understand why they could not … help with practical issues when there were no visible signs [28].
Taking up exercise: ‘personal pride’ or unwelcomed reminders’	“Exercises create pain. You’ll feel the stiffness again. You feel it in your muscles … You don’t really want to be doing them. You don’t want the reminder of how limited you are in some things.” [40] “It’s [not exercising] a lost opportunity and later on that night is when you will dwell on those type of things and all sorts of negative thoughts.” [40]	For some [participants], the ability to exercise to a level that was comparable to peers without AS was a source of personal pride [40]. …these patients were trying out the exercises suggested …and discovering their limitations as a therapy, both in terms of carrying them out in the manner suggested, and benefits for their symptoms. For some, these limitations to the treatments currently offered, combined with a lack of hope for better treatment in the future, seemed to exacerbate their low mood and frustration [25]. In addition to constantly having to take their illness into account, they [participants] believed …they were expected to find the time and energy to follow advice on treatment and exercise. Some of them felt guilty because they did not manage this [33].
Anti-TNF therapy: hope reignited despite concerns	“ …it has made such a big difference already in a relatively short space of time to my life and also mentally as well …Yes, all round, I can go a bit further, do more things, not as tired. Feels like I can conquer the world.” [30] “I think [the condition took] about two months before it started to get better [after being on anti-TNF medication] …I started playing football again within a month and I never thought, never thought I would play football again ever …” [41] “But one thing that does worry me – say if something was to happen and you couldn’t continue with it. That is the psychological thing about it; you think you know if you have to come off it [anti-TNF medication] how would I cope without. […] …to actually have a taste, you know, [of] normality and then go back, would probably be far worse than having not known what it was like …” [30] “I was worried about what I would do if my disease progressed and the symptoms got worse despite the treatment.” [42]	Because of BT [Biologics Therapy] … the men sometimes forgot that they had a chronic disease because they could begin to live a mostly normal life again [28]. Many apprehensions appeared about being on a ‘strong’ treatment for a long period. Many patients believed that there must be a cost in terms of side effects for taking a medication that was very effective. This fear was reinforced by awareness that certain treatments were relatively recent and that there was limited long term experience with them [47].
Journey of acceptance: worry mixed with hope	“But it’s a double-edged sword, really, because getting the diagnosis is helpful and you know where you stand, and when you talk to people they don’t think you are swinging the lead or you are trying to get out of something… but then the flip-side is, oh God, this is me for the rest of my life; it’s not going to go away, it’s not going to go anywhere”. [46] “I realized that I was not a hypochondriac …in that way the diagnosis was liberating” [28]. “I cannot look forward to the future optimistically. I have worries, […]. Will my disease worsen? Will I be bedridden? Will the medications help? I do not know.” [31] “This is really upsetting to me as I think that perhaps my daughters will get this disease because of me and I will have given it to them.” [47] “I have had to rethink how and what I do for a living, and do find full-time work very demanding… However, I have found a job which I really enjoy and feel very lucky to have found a second career which I love.” [37] “I most likely spent approximately 3–4 years on that [the diagnosis], to basically accept that I had this thing” [28].	The other participants also experienced feelings of sadness and discouragement in connection with the diagnosis, but also relief when getting recognition of their pain after years of uncertainty and insecurity [29]. In the time following the diagnosis, they [participants] experienced a period in which they were uncertain about the changes that AS would bring, both in their private and professional lives. […]. They feared that they, within a few years, would be increasingly dependent on help from others to handle their everyday life [28]. Some patients wrote that AS had slowed their pace of living …and took time to enjoy things […] [39].

#### Delayed diagnosis: a barrier to emotional wellbeing

Five studies provided insights into the negative impact of delayed diagnosis on participants mental well-being [24, 25, 28, 29, 46], many of whom had experienced a protracted pre-diagnosis phase. Intrusive symptoms and pain invalidation by healthcare professionals were key triggers of emotional distress, with some participants experiencing symptoms of depression.

I feel confused. My symptoms are strange; they come and go and I am uncertain if I should seek medical help [46]. Pain limits my daily life tasks. I am frustrated, annoyed and worried about the future [28, 29, 46]. When I seek help, I receive contradictory information by way of explanation for my pain [25, 46] and when my symptoms are attributed to ‘just being out of shape’, I feel disheartened and upset [46]. I am frustrated and fed up having to communicate my suffering to others repeatedly, and angry about the time wasted when healthcare professionals fail to recognise my disease [46]. I am stigmatised and become distressed when others perceive me as a malingerer [24, 25, 46]. I feel sad and helpless.

#### Disruptive symptoms: a source of mood swings

Eleven studies informed this theme [24, 28–36, 47]. Some participants living with a backdrop of persistent pain and loss of independence experienced depression and perceived unwelcome changes to their personality because of symptoms. However, for most, it was the unpredictable nature of disruptive symptoms and the sudden and unexpected transition from normality to dependence that triggered mood swings.

The unpredictable nature of my illness frustrates and irritates me [24, 28, 30, 47].

One minute I feel normal, everything is on track and my worry about how my axial SpA will affect me in the future moves to the back of my mind [24, 28, 29, 47]. The next minute, pain strikes, and I feel totally helpless [24, 31]. The pain can send me bonkers [30] and the accompanying sleep disruption causes my mood to swing [32]. When I am in pain like this, I’m frightened about how I will be in the future [31, 47]. Sometimes, when my disease flares, I feel generally unwell. I have a lot of pain and I feel fatigued; my body is out of sync [24, 33]. During these periods, I am irritable, depressed and withdrawn [24, 28, 34, 35]. I feel guilty for being like this [28, 36].

#### Work disability: a loss of self-esteem

This theme, capturing the loss of participant self-esteem resulting from work disability (absenteeism, reduced hours, role change, loss of work and early retirement) and perceived stigma from work colleagues, was underpinned by contributions from twelve  studies [24, 28–32, 35–39, 46]

Work is a struggle [28–30, 36, 37, 46]. Pain and fatigue make it hard for me to do my job and meet the expectations I have of myself and perceive others also have of me [24, 28, 29, 31, 36, 38]. I try and portray a sense of normality but keeping this up is stressful [36]. Sometimes, I chastise myself [29, 36] as I think I should be able to better manage [29, 36, 37].

I feel guilty about my performance at work or if I need to take time off because of my illness [36–38] and that it negatively impacts my work colleagues [31, 38]. When work colleagues infer that I am 'swinging the lead', I feel frustrated and depressed [24]. I fear the effect of my illness on my work ability in the future [24, 36, 37] and I feel vulnerable [37]. I am unsure how much to disclose about my illness to my employer [24, 36]. I lack confidence at work [36, 37].

When I cannot work, I am irritable and depressed [29, 36, 46] as I am not able to support my family financially [28, 46]. My self-esteem is low [28, 29, 39].

#### Obstacles in interpersonal relationships: a trigger of distress

Many studies described the impact of axial SpA on participant relationships, including with children, partners, work colleagues, friends and healthcare professionals. Predominantly negative affect was expressed by individuals, associated with a loss of connection in their interpersonal relations. However, a few studies highlighted strengthened or new connections as a result of living with axial SpA [28, 48]. Twelve studies [24–28, 30, 31, 33, 39, 47–49] contributed to the theme of obstacles in interpersonal relationships: a trigger of distress.

Axial SpA disrupts my relationships. My symptoms make me irritable with work colleagues, my children and my partner [26, 28, 30, 31, 39, 48]. Sometimes I feel unworthy of my partner because of the way I look [39]. When I am irritable with others or if I need support, I feel guilty [48], although if too much support is offered, I feel useless [25, 48].

When my illness prevents me from being the parent, partner or friend I want to be, I am frustrated, angry and sad [26, 31, 33, 49]. I feel guilty [26, 31].

The invisibility of my arthritis is an obstacle in some of my relationships [24, 28, 47]. I become distressed when others question the validity of my symptoms [24, 27]. Some friends have moved on from me as I am no longer able to join in activities because of my illness. I feel withdrawn and sad [28].

#### Taking up exercise: ‘personal pride’ or unwelcomed reminders’

Data informing this theme comes from six studies [24, 25, 28, 33, 40, 41], providing insights into the mixed emotions experienced in relation to taking up exercise. Exercise is a key intervention in managing axial SpA [51] and for some participants engaging in regular regimens, it was a way to reduce symptoms, improve function and boost mental well-being [25, 28, 40]. For others however, a lack of tangible benefits from exercise and the unwelcome reminder of their disease was disheartening and frustrating, and led to avoidance [25, 40, 41].

I take pride in my exercise routine [25, 40] and in knowing that my exercise capabilities are level with those of my peers who don’t have axial SpA [40]. Physical activity lifts my mood [28, 40] and helps me feel more confident to manage my illness [28, 40, 41]. I feel better about myself when I exercise [40].

I fear exercise [25, 40]. It causes me pain and reminds me that I am ill [24, 25, 40]. I am frustrated and disheartened when I don’t see results from my efforts [25, 40]. I feel guilty about not exercising and negative thoughts play on my mind [33, 40]. My self-esteem is low [25, 40].

#### Anti-TNF therapy: hope reignited despite concerns

Five articles captured the emotional impact of initiating biological therapies in adults with axial SpA [28, 30, 41, 42, 47]. Hope associated with the possibility of symptom amelioration was the primary emotion experienced by participants offered biologic therapies, although running alongside were worries about medicine side effects and lack of sufficient benefit from treatment [42]. For participants taking anti-TNF therapy, hope for the future was reignited, as symptoms lessened and valued activities were reclaimed, although for some, new worries and fears centring on potential withdrawal of treatment emerged.

I feel positive and hopeful about anti TNF therapy. Initially, I was anxious and worried about possible adverse effects or that my symptoms would worsen despite treatment; sometimes I felt desperate thinking I had no choice but to take anti-TNF [42]. But the impact of this treatment on my life is huge. I have regained activities I had lost and I have ‘a new chance in life’ [28]. I feel happy and optimistic about the future [28, 30, 41].

I am still concerned about taking such strong medicine for a long time [47] but now I worry more about my treatment being withdrawn as I still remember the physical and mental torture of this disease [30, 47].

#### Journey of acceptance: hope mixed with worry

For many participants, but not all, diagnosis marked the start of a journey of acceptance and was experienced as relief [25, 28, 29], shock [25] and sadness [29]. Initial hope about a way forward was mixed with worry about what the future would bring, influenced by perceptions of physical deformity resulting from AS [28]. This contributed to distress and depression in the period following diagnosis [25, 28, 29]. Most were forced to adapt to the continual presence of AS in their lives – lives that were infused with worry and fear. For some individuals with established disease, hope of being well had dwindled [24, 35], although joyful living experiences were still possible [37, 39]. Thirteen  studies supported this theme [25, 26, 28, 29, 31, 36, 37, 39, 40, 46, 47, 49, 50]. Receiving a diagnosis of axial SpA validates my experiences. I feel relief that others believe me [

But I am worried. Worried a lot of the time as I don’t know what will happen to me. What will I be like in years to come and what will my prospects be? [25, 28, 29, 31, 36, 39, 46, 47]. I fear dependency [28, 39, 47] and that I will pass this disease to my children and grandchildren [25, 26, 47, 49]. I worry that I might damage my back [25] or do myself harm from joining in physical activities [25, 26, 40, 50] and that my disease will worsen despite treatment [31, 46, 47].

On some occasions I feel hopeful. I have slowed down [37, 39] and found new meaning in my life, which brings joy [28, 37, 39].

#### Analytical themes

Two higher order themes focused on the ‘self’ were developed from our cross study reading of emotions associated with experiences of axial SpA. We adopt Charmaz’s understanding of ‘self’—a fairly stable organised set of ‘characteristics, attributes, attitudes and sentiments that a person holds about himself or herself’ ([52], p279)- as the reference point for our analysis.

#### Loss of self: a well of negative affect

The self is formulated through multiple sets of self-made meanings about the social positions an individual occupies in society (identities) and self-evaluations, such as those about individual competence and self-worth (self-esteem) [53]. Any disruption to, or loss of identities (role, group or personal) through the erosion of identity affirming experiences, or reductions in self-esteem through self-perception, perceived appraisal by others, or social comparison processes, has the potential to diminish the self [52, 53].

Many of the studies included in our synthesis describe disrupted role identities due to impactful disease, in particular work and parent role identities [26, 28–31, 36–39, 46–48]. Some participants experienced total loss of worker role identity due to prolonged absenteeism, while others maintained it, but at the expense of important group identities, such as belonging to a sports team or friendship group. Several reflected that all their energy was focussed towards work, leaving ‘nothing…for my family or my friends’ ([36], p1274).

Personal identities, formed from internalised culturally recognised characteristics, were also abraded by axial SpA. The strong man, [28, 29], the socialiser [38, 39, 43] and the ‘good’ patient performing daily exercises [33, 40] disappeared from view. James [24], writing about his experiences of a life with AS, recalls the threat to his personal identity during disease flares.

“Young men want to be active, vibrant, attractive and ambitious. As a young man I felt all these things. Suddenly, without warning pain would strike and I felt immobile, helpless and frustrated” ([24], p200).

This dissonance, between self-identity meanings and self in situation meanings, gives rise to negative emotions [54], which are likely to become more intense and prolonged when multiple identities cannot be verified.

Alongside identity loss, negative self-evaluations of competence and self-worth, mediated through normative performances in social interactions at work, education, during leisure activities and in social circles, affects self-esteem [53]. Low self-esteem associates with reduced positive affect and heightened negative affect in situations of success and failure, mediated through self-relevant emotions, such as shame as highlighted below [55].

Today, I was in an archive, where we had to …find some files. And we had to stand at a computer and find the numbers…and I couldn’t handle it. I had to go [and] sit down…I feel like an idiot ([29], p145).

Even today, I feel bad about getting paid for just staying home. I don’t contribute to anything. And it’s because I was raised to believe a man gets up in the morning to be the breadwinner for his family ([28], p35).

#### Repair of self: a spring of positive affect

Maintenance and enhancement of self is a key individual concern [56]. Several studies highlighted the positive affect experienced by participants when self was repaired following efficacious treatment with biologic therapy [28, 30, 41], largely through re-establishing old identities as participants once again engaged in meaningful work and family activities [30] and resumed old hobbies [41].

For those not on biologic therapy, positive affect was evident when connections with pre-illness self could be maintained. Adaptive coping, for example, through adjustment of activity patterns and ambitions, enabled some participants to engage in other pursuits that made them happy [37, 39], while self-appraisal of work attendance compared to the records of healthy others [24, 39], re-enforced self-worth.

### Social media review

Extracted posts (*n*=537) came from the NASS community forum (*n*=474); ASone, an online platform for younger adults with axial SpA (*n*=38); and Faces of AS (*n*=25), a website hosting stories of people living with AS. Posts were from people with varying symptom onset duration, time since diagnosis, disease-related disability and comorbidity, and treatment regimens, and captured experiences and opinions relating to living with axial SpA. Nearly half (48%) of all posts were from women.

Data from posts, for the most part, supported the seven themes developed from the qualitative evidence synthesis, although some emotions and contexts were not identified in posts, for example, fear of exercise because of anticipatory pain and guilt at non-compliance with exercise regimens. Accounts in posts were often fuller than those extracted from published papers and several additional constructs, reflecting the contemporaneous nature of these data, were identified. Two of the themes formulated through the evidence synthesis were broadened (work disability: a loss of self-esteem and anti-TNF: hope re-ignited despite concerns), and a new theme – COVID-19: uncertainty and anxiety during the pandemic, was developed.

#### Work disability: a loss of self-esteem

Work disruption, such as absenteeism and having to work reduced hours due to illness was reported frequently in posts, although several individuals highlighted career fulfilment despite their condition and experienced pride in their workplace attendance. Negotiating reduced working hours, claiming or considering claiming disability and sickness benefits was a source of stress, frustration and worry for some.

Seeking financial benefits because I cannot work is a degrading process [NASS forum 287, 288]. I fear the PIP (Personal Independence Payment) assessments [NASS forum 256, 274, 275, 286]. I am frustrated and upset when authorities do not recognise my disability and my claims are denied [NASS forum 264, 270, 273, 295].

#### Anti-TNF: hope re-ignited despite concerns

While many posts referred to benefit derived from taking anti TNF therapy, several individuals highlighted the waning impact of biologic therapy on them over time and the need to stop treatment because of adverse effects. As in our evidence synthesis, some expressed worry and fear about anti-TNF side effects (specifically increased infection risk) and symptom recurrence. Additionally, injection / needle fear, both in people due to start anti-TNF and those on treatment, and dissatisfaction about an actual or planned switch to a biosimilar, were expressed.

I fear my biologic injections [Faces of AS 9; NASS forum 32-34, 192]. The thought of the needle makes me anxious, and it is a mental barrier that is hard to overcome [NASS forum 32-34, 192]

I am unhappy being switched from my usual biologic therapy to a biosimilar [NASS forum 190, 192, 220, 221] as my AS symptoms have returned [NASS 190, 191, 220].

#### COVID-19: uncertainty and anxiety during the pandemic

Thirty-five posts from 23 individuals informed the generation of this theme, capturing emotions expressed during the UK’s first wave of the coronavirus pandemic and national lockdown. Common triggers for emotions were administration of biologic injections and shielding in the context of immunosuppression, and reduced access to rheumatology services.

I am uncertain and anxious. I don’t know whether or not to take my usual biologic injections and risk becoming unwell with COVID-19 or go without and suffer symptoms [NASS forum 147, 149, 150, 151, 154, 324, 336, 337]. The information I receive about how much I should shield is inconsistent and I am unsure and confused [NASS forum 13-15, 258-261, 324, 338-341]. I feel alone, unsupported and unmotivated [NASS forum 317, 320, 446, 447, 462, 468, 469].

COVID-19 is preventing the start of treatment for my axial SpA. I know NHS staff are busy with the pandemic but I am frustrated [325, 449]. Living like this is taking a toll on my mental health [ASone 16, NASS 152, 445, 460, 461].

## Discussion

Conceived through a lens of emotionality, this qualitative evidence synthesis and the findings from our social media review supplement prevalence and incidence data on mental ill health among people with axial SpA. The majority of participants contributing data to our evidence synthesis were men, most likely reflecting traditional classification criteria that emphasised radiographic sacroiliitis. In contrast, posts selected from our review of social media show a more balanced gender representation. This latter finding may reflect known associations between female gender and online health information seeking [57, 58], or similarities between men and women in communication styles and the nature of messages (informational or emotional) posted to mixed gender online health communities [59, 60].

Overall, our data reveal a preponderance of negative affect experienced in response to living with axial SpA. We did not identify any notable gender differences in affect within our themes, although the contribution of four high quality richly descriptive studies focused solely on male participants, illuminates a profound impact of axial SpA on men’s emotional health. The negative affect discerned in this study is conditioned through existing and anticipated symptoms, and appraisals and attributions of events (real or perceived), including failed expectations, stigma, non-verification of identities and loss of self-esteem.

These findings map to the affective experiences of other populations living with inflammatory arthritis. Narratives from qualitative evidence syntheses capturing the lived experiences of people with RA [12, 61, 62], inflammatory arthritis [63], PsA [14], Spondyloarthritis [62] and Gout [13] are similarly permeated with negative emotions: frustration, anger and hopelessness associated with suffering and unfolding social interactions [12–14, 61, 63]; pain-related fear [13, 14, 63]; anger and guilt in response to task and goal progress [12, 14, 61–63]; fear over prospects [12, 14, 62, 63]; and sadness at perceptions of loss [12–14]. The uncertainty and anxiety during the early phase of the pandemic identified in our social media review echoes findings from other studies exploring pandemic experiences among people with rheumatic and musculoskeletal disorders [64, 65]. A sentiment analysis of tweets in English posted by people with arthritis between March and April 2020, highlights individual concern about the health impact of SARS-CoV-2 and confusion over health messages received [64].

The emotions and their sources captured in this review are not surprising given the disease-related burden experienced by people living with axial SpA [66] and established links between inflammation and depression [67]. Yet, the relevance of emotions in people with inflammatory arthritis gains prominence if we consider the bi-directional relationships between emotions and inflammatory response [68, 69], emotions and perceptions of pain [70] and the role of affect in self-care behaviors [71, 72]. Additionally, lower positive affect reactivity and lingering negative affect in response to daily life stressors is influential in predicting mental and physical ill-health [11, 73, 74].

Several studies have identified associations between higher cytokine levels and momentary and recalled negative affect [68, 69, 75]. Perceptions of pain, integral to decisions about treatment of axial SpA, are heightened under conditions such as anxiety [76], sadness and anger [77]. Furthermore, while studies investigating the association between negative emotions and physical activity (known to be suboptimal in people with axial SpA [78, 79] are limited [71], negative emotions are a recognised barrier to medicines use [72, 80] and potentially contribute to the variability in medicines adherence seen among people with axial SpA [81]. Fears about ‘inevitable’ adverse effects from long-term biologic therapy (despite no treatment side effects) have been reported by patients with rheumatic diseases identified as poorly adherent to treatment [82]. Similar concerns, about side effects, are also held by patients considering switching from a bio-originator to a biosimilar [83, 84], despite comparable safety and efficacy data [85]. In a cohort of 96 patients taking a bio-originator for their rheumatic disease, concern about switching to a biosimilar was augmented in those experiencing a strong emotional response to their condition [84]. Thus, in the context of a heightened emotional burden, patients may show reluctance to switch, or experience a nocebo effect if they do switch [86], with implications for adherence to, and persistence with, biosimilars [87].

Notwithstanding the pervasiveness of negative affect identified in this review, effective interventions such as psychological treatments (e.g. cognitive behavioural therapy) and physical activity, can reduce psychological distress in people with inflammatory arthritis [88–92]. Alongside these, peer support programmes may facilitate coping in the early stages following diagnosis [93] and promote successful adjustment to living with axial SpA, with the potential for better health outcomes and quality of life.

### Strengths and limitations

The findings of this review need to be considered in light of its strengths and weaknesses. Key strengths include a novel focus on emotional wellbeing among people with axial SpA, a wide-ranging search of formal and grey literature for eligible studies from which to develop a qualitative evidence synthesis, and rigorous processes of data extraction and critical appraisal. Additionally, our review of social media extends insights gained from our evidence synthesis though its contemporality, and addresses in part, the gender bias evident in our synthesis.

However, our review also has limitations. First, inter-assessor reliability of study selection, based on an initial screen of records was moderate [94]. While the reviewers discussed screening decisions and clarified selection criteria in an effort to improve inter-assessor reliability, we did not repeat dual screening of records due to resource limits and so we do not know if reliability improved. Second, under half of the studies included in our review contained thick qualitative data. We explored different approaches to data synthesis (e.g. meta-ethnography) and decided, given the nature of our data, that a thematic synthesis was the most appropriate. Finally, we acknowledge the potential influence of sociocultural factors and gender on appraisal of events and the experience and expression of emotion [95–97], the limitations of cross-sectional data, and written text in communicating the dynamic nature of emotional experiences over time [98].

## Conclusion

Our study provides an opportunity to ‘see’ emotions and their sources in the lives of people with axial SpA and complements data from quantitative studies about the prevalence and incidence of psychological states in this population. Our synthesis illuminates the negative affect associated with symptoms, failed expectations and loss of self, both pre-and post- diagnosis, in a predominantly male population with Ankylosing Spondylitis, most of whom were not taking biologic therapy. The under representation of women in our review is noteworthy. Thus, we have undertaken a contemporary primary qualitative study to explicitly explore affect in both men and women with axial SpA in the early phase after diagnosis. This study is currently in its final stages.

### Supplementary Information

**Additional file 1.** Search strategies.

**Additional file 2.** Study quality assessment.

**Additional file 3.** Checklist for reporting the synthesis of qualitative research.

## Data Availability

Data used in our qualitative evidence synthesis were extracted from published manuscripts identified in our systematic review. The dataset used and analysed in our social media review is not publically available as permission to share participant personal data was not sought.
