# Supplementary material for: Exploring the emotional impact of axial Spondyloarthritis: a systematic review and thematic synthesis of qualitative studies and a review of social media
Source: BMC Rheumatol. 2023 Aug 23;7:26. doi: 10.1186/s41927-023-00351-w (PMC10464274; doi:10.1186/s41927-023-00351-w)
Supplement: Supplementary file 1 — Additional file 1. Search strategies. [file 41927_2023_351_MOESM1_ESM.pdf]

Additional file 1

Search strategies (originally run in December 2019, updated 22nd March 2021)

Original search
Database: Ovid MEDLINE(R) ALL <1946 to December 04, 2019>
----
1 Spondylitis, Ankylosing/ or Spondylitis/
2 (ankylose* or spondyl* or SpA or bekhterev* or bechterew*).mp.
3 1 or 2
4 "Quality of Life"/
5 (quality of life or qol).mp.
6 4 or 5
7 exp Mental Health/
8 (Mental health or emotional?wellbeing or psychological well?being).mp.
9 7 or 8
10 Life Change Events/
11 Affective Symptoms/
12 Depression/
13 depressive disorder/ or depressive disorder, major/ or dysthymic disorder/
14 exp Anxiety/ or exp Anxiety Disorders/
15 exp Stress, Psychological/
16 exp Patient Satisfaction/
17 (depress* or depressive disorder* or dysthymic disorder* or anxiety or anxiety disorder* or psychological stress or anger or distress or patient satisfaction).mp.
18 6 or 9 or 10 or 11 or 12 or 13 or 14 or 15 or 16 or 17
19 3 and 18

Database: Embase Classic+Embase <1947 to 2019 December 06>
----
1 spondylitis/ or ankylosing spondylitis/ or spondylarthritis/
2 (ankylose* or spondyl* or SpA or bekhterev* or bechterew*)
3 1 or 2
4 "quality of life"/
5 (quality of life or qol).mp.
6 4 or 5
7 exp mental health/
8 (mental health or emotional?wellbeing or psychological well?being).mp.
9 7 or 8
10 life event/
11 emotional disorder/
12 depression/
13 anxiety/ or anxiety disorder/
14 mental stress/
15 patient satisfaction/
16 (depress* or depressive disorder* or dysthymic disorder* or anxiety or anxiety disorder* or psychological stress or anger or distress or patient satisfaction)
17 6 or 9 or 10 or 11 or 12 or 13 or 14 or 15 or 16
18 3 and 17

Database: Cinahl

----
1 MH Spondylitis or MH Ankylosing Spondylitis or MH Spondylarthritis/
2 (ankylose* or spondyl* or SpA or bekhterev* or bechterew*)
3 1 or 2
4 MH "Quality of Life"
5 (quality of life or qol).mp.
6 4 or 5
7 MH Mental Health
8 (mental health or emotional?wellbeing or psychological well?being).mp.
9 7 or 8
10 MH Life Change Events+
11 Affective Symptoms/
12 MH Depression or MH Depression, Reactive or MH Dysthymic Disorder
13 MH Anxiety or MH Anxiety Disorders/
14 MH Stress, Psychological
15 MH Patient Satisfaction
16 (depress* or depressive disorder* or dysthymic disorder* or anxiety or anxiety disorder* or psychological stress or anger or distress or patient satisfaction)
17 6 or 9 or 10 or 11 or 12 or 13 or 14 or 15 or 16
18 3 and 17

Database: PsycINFO <1806 to December Week 2 2019>
----
1 arthritis/
2 (ankylose* or spondyl* or SpA or bekhterev* or bechterew*).mp.
3 1 or 2
4 exp "Quality of Life"/
5 (quality of life or qol).mp.
6 4 or 5
7 exp Mental Health/
8 (Mental health or emotional?wellbeing or psychological well?being).mp
9 7 or 8
10 exp Life Experiences/ or exp Life Changes/
11 affective disorders/
12 major depression/ or dysthymic disorder/ or reactive depression/
13 exp Anxiety Disorders/ or exp Anxiety/
14 exp Psychological Stress/
15 exp Client Satisfaction/
16 (depress* or depressive disorder* or dysthymic disorder* or anxiety or anxiety disorder* or psychological stress or anger or distress or patient satisfaction).mp.
17 6 or 9 or 10 or 11 or 12 or 13 or 14 or 15 or 16
18 3 and 17

Web of Science core collection search
----

1. ankylose* or spondyl* or SpA or bekhterev* or bechterew*

2. depress* or depressive disorder* or dysthymic disorder* or anxiety or anxiety disorder* or psychological stress or anger or distress or patient satisfaction

3. 1 AND 2

Scopus
----

1. ankylose* or spondyl* or SpA or bekhterev* or bechterew*

2. depress* or “depressive disorder*” or “dysthymic disorder*” or anxiety or “anxiety disorder*” or “psychological stress” or anger or distress or “patient satisfaction”

3. 1 AND 2

Grey literature & systematic reviews

Ethos search:

1. ankylose* or spondyl*

OpenGrey search:

1. ankylose* or spondyl*

Cochrane search:

1. ankylose* or spondyl* or SpA or bekhterev* or bechterew*

UPDATED SEARCH: week of 15th March 2021

Database: Ovid MEDLINE(R) ALL <1946 to March 26, 2021>
----

1 spondylitis/ or spondylitis, ankylosing/

2 (ankylose* or spondyl* or SpA or bekhterev* or bechterew*).mp.

3 1 or 2

4 exp "Quality of Life"/

5 (quality of life or qol).mp.

6 4 or 5

7 exp Mental Health/

8 (Mental health or emotional?wellbeing or psychological well?being).mp

9 7 or 8

10 Life Change Events/

11 Affective Symptoms/

12 Depression/

13 depressive disorder/ or depressive disorder, major/ or dysthymic disorder/

14 exp Anxiety/ or exp Anxiety Disorders/

15 exp Stress, Psychological/

16 exp Patient Satisfaction/

17 (depress* or depressive disorder* or dysthymic disorder* or anxiety or anxiety disorder* or psychological stress or anger or distress or patient satisfaction).mp.

18 6 or 9 or 10 or 11 or 12 or 13 or 14 or 15 or 16 or 17

19 3 and 18

20 limit 19 to yr="2020 - 2021"

Database: Embase <1974 to 2021 Week 12>
----

1 spondylitis/ or ankylosing spondylitis/ or spondylarthritis/

2 (ankylose* or spondyl* or SpA or bekhterev* or bechterew*).

3 1 or 2

4 exp "quality of life"/

5 (quality of life or qol).mp.

6 4 or 5

7 exp mental health/

8 (Mental health or emotional?wellbeing or psychological well?being).mp.

9 7 or 8

10 life event/

11 emotional disorder/

12 depression/

13 depressive disorder/ or depressive disorder, major/ or dysthymic disorder/

14 anxiety/ or anxiety disorder/

15 mental stress/

16 patient satisfaction/

17 (depress* or depressive disorder* or dysthymic disorder* or anxiety or anxiety disorder* or psychological stress or anger or distress or patient satisfaction).mp.

18 6 or 9 or 10 or 11 or 12 or 13 or 14 or 15 or 16 or 17

19 3 and 18

20 limit 19 to yr="2020 - 2021"

Database: APA PsycInfo <1806 to March Week 4 2021>
----

1 arthritis/

2 (ankylose* or spondyl* or SpA or bekhterev* or bechterew*).mp

3 1 or 2

4 exp "Quality of Life"/

5 (quality of life or qol).mp.

6 4 or 5

7 exp Mental Health/

8 (Mental health or emotional?wellbeing or psychological well?being).mp.

9 7 or 8

10 exp Life Experiences/ or exp Life Changes/

11 affective disorders/

12 major depression/ or dysthymic disorder/ or reactive depression/

13 exp Anxiety Disorders/ or exp Anxiety/

14 exp Psychological Stress/

15 exp Client Satisfaction/

16 (depress* or depressive disorder* or dysthymic disorder* or anxiety or anxiety disorder* or psychological stress or anger or distress or patient satisfaction).mp.

17 6 or 9 or 10 or 11 or 12 or 13 or 14 or 15 or 16

18 3 and 17

19 limit 18 to yr="2020 - 2021"

Cinahl

S1. MH Spondylitis or MH Ankylosing Spondylitis or MH Spondylarthritis/
S2. ankylose* or spondyl* or SpA or bekhterev* or bechterew*
S3. S1 OR S2
S4. MH "Quality of Life"
S5. quality of life or qol
S6. S4 OR S5
S7. MH Mental Health
S8. mental health or emotional?wellbeing or psychological well?being
S9. S7 OR S8
S10. MH Life Change Events+
S11. MH "Affective Symptoms"
S12. MH Depression or MH Depression, Reactive or MH Dysthymic Disorder
S13. MH Anxiety or MH Anxiety Disorders/
S14. MH Stress, Psychological
S15. MH Patient Satisfaction

S16. depress* or depressive disorder* or dysthymic disorder* or anxiety or anxiety disorder* or psychological stress or anger or distress or patient satisfaction
S17. S6 OR S9 OR S10 OR S11 OR S12 OR S13 OR S14 OR S15 OR S16
S18. S3 AND S17
S19. Limit to publication years 2020 & 2021

Web of Science

----

1. ankylose* or spondyl* or SpA or bekhterev* or bechterew*

2. depress* or depressive disorder* or dysthymic disorder* or anxiety or anxiety disorder* or psychological stress or anger or distress or patient satisfaction

3. 1 AND 2

4. Refine by publication years 2020, 2021

Scopus

----

1. ankylose* or spondyl* or SpA or bekhterev* or bechterew*

2. depress* or depressive disorder* or dysthymic disorder* or anxiety or anxiety disorder* or psychological stress or anger or distress or patient satisfaction

3. 1 AND 2

4. Refine to publication years 2020,2021

Grey literature and systematic review

Ethos search:

1. ankylose* or spondyl*

OpenGrey search:

1. ankylose* or spondyl*

Cochrane search:

1. ankylose* or spondyl* or SpA or bekhterev* or bechterew*
2. depress* or depressive disorder* or dysthymic disorder* or anxiety or anxiety disorder* or psychological stress or anger or distress or patient satisfaction
3. #1 and #2
