# Supplementary material for: Exploring the emotional impact of axial Spondyloarthritis: a systematic review and thematic synthesis of qualitative studies and a review of social media
Source: BMC Rheumatol. 2023 Aug 23;7:26. doi: 10.1186/s41927-023-00351-w (PMC10464274; doi:10.1186/s41927-023-00351-w)
Supplement: Supplementary file 2 — Additional file 2. Study quality assessment. [file 41927_2023_351_MOESM2_ESM.pdf]

Additional file 2.

Study quality assessment using a modified version of the Critical Appraisal Skills Programme (CASP) tool

First author (year)	Was there a clear statement of the aims of the research?	Is a qualitative methodology appropriate?	Was the research design appropriate to address the aims of the research?	Are the study's theoretical underpinnings (e.g. ontological and epistemological assumptions; guiding theoretical framework(s)) clear consistent and conceptually coherent?	**Was the recruitment strategy appropriate to the aims of the research?**<br>*i) Explained how participants were selected?*<br>*ii) Explained why participants were most appropriate*<br>*iii) Any discussions around recruitment*	Was the data collected in a way that addressed the research issue?	Has the relationship between researcher and participants been adequately considered?	Have ethical issues been taken into consideration?	**Was the data analysis sufficiently rigorous?**<br>*i) In-depth description of analysis process?*<br>*ii) clear how categories/themes derived from the data*<br>*iii) explanation about how data presented were selected*<br>*iv) sufficient data presented to support findings*<br>*v) contradictory data taken into account*<br>*vi) Critical examination of researchers own role*	Is there a clear statement of findings?	How valuable is the research?
**Mengshoel** (2010)	Yes	Yes	Yes	Can't tell	Partly i), ii)	Yes	Partly	Yes	Yes i), ii), iii), iv), v), vi)	Yes	Valuable
**Martindale** (2014)	Yes	Yes	Yes	Yes	Partly i), ii),	Yes	Can't tell	Yes	Yes i), ii), iii), iv), v), vi	Yes	Valuable
**O'Dwyer** (2016)	Yes	Yes	Yes	Yes	Partly i), ii),	Yes	Yes	Yes	Yes i), ii), iii), iv), v), vi	Yes	Valuable
**Stockdale** (2008)	Yes	Yes	Yes	Yes	Yes i), ii), iii)	Yes	Yes	Yes	Partly i), ii), iv), v), vi)	Yes	Valuable
Davies (2013)	Yes	Yes	Yes	Can't tell	Yes i), ii), iii)	Yes	Can't tell	Yes	Partly i), ii), iii), iv), v)	Yes	Valuable
Raybone (2019)	Yes	Yes	Yes	Somewhat	Yes i), ii), iii)	Yes	Somewhat	Yes	Partly i), ii), iv), v), vi)	Yes	Valuable
**Lacaille** (2007)	Yes	Yes	Yes	Somewhat	Yes i), ii), iii)	Yes	Can't tell	Partly	Partly i), ii), iii), iv)	Yes	Valuable
Stockdale (2014)	Yes	Yes	Yes	Yes	Partly i), iii)	Yes	Yes	Yes	Partly i), ii), iv), v), vi)	Yes	Valuable
**Barlow** (1999)	Yes	Yes	Yes	Can't tell	Partly i), ii)	Yes	Can't tell	Can't tell	Partly i), ii), iv), v)	Yes	Valuable

**Barlow** (2001)	Yes	Yes	Yes	Can't tell	Partly i), ii)	Yes	Can't tell	Can't tell	Partly i), iii), iv), v)	Yes	Valuable
**Hamilton-West** (2009)	Yes	Yes	Yes	Can't tell	Partly i), iii)	Yes	Can't tell	Somewhat	Partly i), ii), iv), v)	Yes	Valuable
Farren (2013)	Yes	Yes	Yes	Yes	Partly i), ii)	Yes	Yes	Yes	Partly i), ii), v), vi)	Yes	Valuable
**Primholdt** (2016)	Yes	Yes	Yes	Yes	Partly i), ii)	Yes	Somewhat	Yes	Partly i), ii), iii), iv)	Yes	Valuable
**Madsen** (2015)	Yes	Yes	Yes	Somewhat	Partly ii), iii)	Yes	Can't tell	Yes	Partly i), ii) n/a, iv)	Yes	Valuable
**Berenbaum** (2014)	Yes	Yes	Yes	Can't tell	Partly i), ii)	Yes	Can't tell	Can't tell	Partly iii), iv	Somewhat	Valuable
**Bagcivan** (2015)	Yes	Yes	Yes	Yes	Partly ii)	Yes	Can't tell	Yes	Partly i), ii), iv), vi)	Yes	Valuable
Kwan (2019)	Yes	Yes	Yes	Can't tell	Partly i), ii)	Yes	Can't tell	Yes	Partly i), ii) n/a iii), iv),	Yes	Valuable
Connolly (2019)	Yes	Yes	Yes	Can't tell	Partly i), iii)	Yes	Can't tell	Yes	Partly i), ii), iv)	Yes	Valuable
Brophy (2013)	Yes	Yes	Yes	Can't tell	Partly i), iii)	Somewhat	Can't tell	Yes	Partly i), iv)	Somewhat	Valuable
Hwang (2020)	Yes	Yes	Yes	Can't tell	Partly i), ii)	Yes	Can't tell	Yes	Partly i), iii)	Yes	Valuable
Haugli (2004)	Yes	Yes	Yes	Can't tell	Partly i)	Yes	Partly	Somewhat	Partly ii), iii), iv)	Yes	Valuable
Cury (1995)	Yes	Yes	Yes	Can't tell	Partly i)	Somewhat	Can't tell	Can't tell	Partly i)	Yes	Valuable
Brophy (2002)	Yes	Yes	Somewhat	Can't tell	Partly i)	Yes	Can't tell	Somewhat	Can't tell	Yes	Valuable
Boonen (2009)	Yes	Yes	Somewhat	Can't tell	Partly i)	Yes	Can't tell	Can't tell	Partly i)	Yes	Yes
Cinar (2014)	Yes	Yes	Somewhat	Can't tell	Partly Ii), iii)	Yes	Can't tell	Yes	Can't tell	Yes	Yes

Studies with thick descriptive data are indicated in **Bold**

Grey literature quality assessment using the Authority, Accuracy, Coverage, Objectivity, Date, Significance (AACODS) checklist

First author (year)	**Authority**<br>Individual author<br><br>Associated with a reputable organisation?<br>Professional qualifications or considerable experience?<br>Produced/published other work (grey/black) in the field?<br>Recognised expert, identified in other sources?<br>Cited by others?<br>Higher degree student under "expert" supervision?<br><br>Does the item have a detailed reference list or bibliography?	**Accuracy**<br><br>Does the item have a clearly stated aim or brief? If so, is this met?<br>Does it have a stated methodology? If so, is it adhered to?<br>Has it been peer-reviewed?<br>Has it been edited by a reputable authority?<br>Supported by authoritative, documented references or credible sources?<br>Is it representative of work in the field? If No, is it a valid counterbalance?<br>Is any data collection explicit and appropriate for the research?<br>Is it an accurate, unbiased interpretation or analysis?	**Coverage**<br><br>Are any limits clearly stated?	**Objectivity**<br><br>Is the author's standpoint clear?<br>Does the work seem to be balanced in presentation?	**Date**<br><br>Does the item have a clearly stated date related to content?<br>Has key contemporary material been included?	**Significance**<br><br>Is the item meaningful?<br>Does it add context?<br>Does it enrich or add something unique to the research?<br>Does it strengthen or refute a current position?<br>Would the research area be lesser without it?<br>Is it integral, representative, typical?<br>Does it have impact?
**James** (2009)	Yes	Yes	No	Yes	Yes	Yes
Thompson (2011)	Yes	Yes	Yes	Yes	Yes	Yes

Studies with thick descriptive data are indicated in **Bold**
