# Supplementary material for: Exploring the emotional impact of axial Spondyloarthritis: a systematic review and thematic synthesis of qualitative studies and a review of social media
Source: BMC Rheumatol. 2023 Aug 23;7:26. doi: 10.1186/s41927-023-00351-w (PMC10464274; doi:10.1186/s41927-023-00351-w)
Supplement: Supplementary file 3 — Additional file 3. Checklist for reporting the synthesis of qualitative research. [file 41927_2023_351_MOESM3_ESM.pdf]

Additional file 3.

Checklist for reporting the synthesis of qualitative research (modified from Tong et al. [22])

No	Item	Explanation
1	Aim	The study research question is reported.
2	Synthesis methodology	A thematic synthesis was employed due to insufficient data from which to develop third order interpretations necessary for meta ethnography.
3	Approach to searching	A pre-planned comprehensive search strategy was developed to seek all available studies
4	Inclusion criteria	Specified.
5	Data sources	Data sources described and the rationale for use are provided.
6	Electronic search strategy	Search strategies are presented in Supplementary file 1.
7	Study screening methods	Described in methods section.
8	Study characteristics	Presented in Table 1.
9	Study selection results	Presented in Figure 1.
10	Rationale for appraisal	Studies were appraised based on their description of an appropriate recruitment strategy and rigour of data analysis.
11	Appraisal items	Appraisal tools and criteria are stated.
12	Appraisal process	The number of reviewers who undertook critical appraisal and the process of moderation in case of disagreement, are stated.
13	Appraisal results	Presented in Supplementary file 1.
14	Data extraction	Relevant text in the abstract, results, discussion and supplementary files were analysed.
15	Software	NVivo 12 (QSR International) was used for data management and coding.
16	Number of reviewers	The number of reviewers involved in coding and analysis is stated.
17	Coding	The coding process is described.
18	Study comparison	The process of study comparison is described.
19	Derivation of themes	Descriptive themes were derived deductively. Analytical themes were derived via deduction and induction.
20	Quotations	Quotes from participants and authors interpretations are presented in Table 2.
21	Synthesis output	Useful results that go beyond a summary of the primary studies are presented and limitations of the synthesis are discussed.
